# Blending of the Thermodynamically Incompatible Polyvinyl Chloride and High-Pressure Polyethylene Polymers Using a Supercritical Fluid Anti-Solvent Method (SEDS) Dispersion Process

**DOI:** 10.3390/polym15091986

**Published:** 2023-04-22

**Authors:** Vener F. Khairutdinov, Ilnar Sh. Khabriev, Farid M. Gumerov, Rafail M. Khuzakhanov, Ruslan M. Garipov, Lenar Yu. Yarullin, Ilmutdin M. Abdulagatov

**Affiliations:** 1 Chemical Engineering Department, Kazan National Research Technological University, 420015 Kazan, Russia; 2 Department of Physical and Organic Chemistry, Dagestan State University, 367008 Makhachkala, Russia; 3 Geothermal and Renewal Energy Institute of the High Temperature Joint Institute of the Russian Academy of Sciences, 367015 Makhachkala, Russia

**Keywords:** polyvinyl chloride, high-pressure polyethylene, диcпepгиpoвaниe, supercritical CO_2_, SEDS method

## Abstract

The experimental solubility data of polyvinyl chloride (PVC) and high-pressure polyethylene (HPPE) in organic solvents (toluene, dichloromethane, and chloroform) at temperatures ranging from 308.15 to 373.15 K at atmospheric pressure are reported in the present paper. The solubility of the polymers (PVC and HPPE) in organic solvents (toluene, dichloromethane, and chloroform) was studied at temperatures between 298 and 373 K. The supercritical SEDS dispersion of PVC and HPPE polymer blends at pressures between 8.0 and 25 MPa and at temperatures from 313 to 333 K are reported in the present work. The kinetics of crystallization and phase transformation in polymer blends obtained by blending in a melt, and using the supercritical SEDS method, have been studied. The effect of the HPPE/PVC ratio on the thermal and mechanical characteristics of the polymer blends has been studied. For all studied polymer blends and pure polymers obtained using the SEDS method, the heat of fusion Δ_fus_*H* exceeds the values obtained by blending in the melt by 1.5 to 5) times. The heat of fusion of the obtained polymer blends is higher than the additive value; therefore, the degree of crystallinity is higher, and this effect persists after heat treatment. The relative elongation decreases for all polymer blends, but their tensile strength increases significantly.

## 1. Introduction

The blending of thermodynamically incompatible polymers provides a powerful way to obtain materials with improved physical and mechanical properties. However, most blended polymers are thermodynamically immiscible, and compatibilization is required to obtain a maximum combined effect greater than the sum of their separate effects. Several excellent reviews on the compatibilization of polymer blends exist (see, for example, [1,2]). Polymer blending is a great way to develop new polymeric materials which combine the excellent properties of pure components. Polymer blend-based materials with advanced properties have increasing applications in the industry such as automotive, electrical and electronic, packaging, building and household, etc. Most polymer blends are fully immiscible. They have a sharp interface, and the adhesion between both blend phases is poor; therefore, these blends are useless without compatibility. An example of a fully immiscible blend is the HPPE/PVC polymer blend. As is known [3], PE and PVC are completely incompatible polymers. This blend has become commercially successful only after being efficiently compatibilized.

The development of the industry constantly requires the creation of new polymeric materials with higher performance characteristics. One of the ways to solve this problem is the use of polymer blends, especially based on well-known materials such as polyethylene (PE) and polyvinyl chloride (PVC), which are high-consumption materials.

At the same time, it should be noted that the waste of these polymers is also quite large, and the problem of their recycling is an urgent task. Since PE and PVC are completely incompatible polymers [3], the production of blends based on PE and PVC is rather problematic. In this regard, a lot of research has been carried out to improve the compatibility of these polymers, and most of the work is devoted to the production of blends from the waste of these polymers. Blends of the different types of polymers combine some of the important characteristics of both blend constituents.

There are three main methods for increasing (enhancing) the compatibility of these polymers [4]: (1) the addition of a block copolymer (copolymerization) having a similarity for both blend polymers; (2) the addition of reactive polymers to the blend; and (3) the addition of low molecular weight additives interacting with both blend components. Sharshir et al. [5] reported PVC and HPPE blends compatibilization by gamma irradiation in the dose of 0, 10 and 20 kGy. They showed remarkable improvement in mechanical properties after the gamma irradiation process. For example, the elongation is raised by 21% to 52% by increasing the SBR content between 1% to 3%. The stress-strain behavior and morphology of partially compatible PVC/CPE blends were studied by Zhang and Peixin [6]. The mechanical and thermal properties of an uncompatibilized blend of (PVC/HDPE) were studied by Maou et al. [7]. They showed that MAH formed bridges between the PVC and HDPE polymers improved adhesion between the two immiscible polymers, increased the initial thermal degradation temperature of the blend at approximately 31 °C, and increased the glass transition temperature. Thus, the addition of MAH into the PVC/HDPE blend enhances the thermodynamic compatibility of the blend. The reactive interfacial agent (PCL-g-GMA) for PCL-starch blends is synthesized using supercritical CO_2_ as a reaction medium. Iqbal et al. [8] found that reaction efficiency in supercritical CO_2_ is much better than in the melt. Moreover, less degradation occurred for samples produced in supercritical CO_2_. Additionally, the use of the PCL-g-GMA made in scCO2 as an interfacial agent in a ternary blend of PCL/starch/PCL-g-GMA exhibits better mechanical properties in comparison with those prepared in the melt. The review by Graziano et al. [9] summarizes the progress and future perspectives in compatibilization techniques for enhancing the interfacial adhesion between PE and PP. Moreover, the authors provided a comprehensive discussion of the influence of thermodynamics on the PE/PP interface, which allows the blend to be used for commercial applications. Knez et al. [10] reviewed the application of supercritical fluids as processing media for particle formation processes, and presented recent advances and trends in development. Montes et al. [11] studied the effect of operation parameters of the supercritical antisolvent process, such as temperature, pressure, and concentration of solution polymer blends ratio on particle size distribution. Supercritical fluids as a processing solvent in polymer applications such as polymer modification, formation of polymer composites, polymer blending, microcellular foaming, and particle production have been studied by Nalawade et al. [12]. Rossmann et al. [13] studied the supercritical antisolvent technology to crystallize paracetamol particles. Supercritical CO_2_ has been used as an antisolvent, while ethanol and acetone and their mixtures are used as solvents. They found that by varying the content of ethanol between 1% and 5 wt% in the solution, it is possible to adjust the structure of the produced particles (crystals). The authors illustrated that particles with defined properties could be achieved by the conventional operating parameters, such as pressure, temperature, and solute concentration, and the flow rate ratio of solution, type of solvent, and antisolvent, adjusted during the process.

The recycling of PVC/PE blend wastes has been studied in [14]. In this work, the graft copolymer of chlorinated polyethylene with polymethyl acrylate (CPE-graph-PMA) was used as a modifier for the PVC/LPPE, which was added in an amount of 1.5%, and formed stable blends with both PVC and LPPE. An increase in the compatibility of PVC with HPPE in the melt, with the addition of polyethylene into the blend from 2 to 5%, is explained by the improvement in physical and mechanical properties.

Based on the brief review publication in the field, several points can be highlighted. First, all the studied blends were obtained in the traditional way, namely, by blending in the melt. Secondly, the concentration of one of the components was no more than 10%. Thirdly, small additions of other components have been used as compatibilizers.

Supercritical fluids can be used for the formation of polymer blends with unique properties for use in different applications. In our several previous publications [15,16,17], we have successfully used the supercritical SEDS technique to blend thermodynamically incompatible polymers such as ethylene vinyl acetate copolymer/polycarbonate [15], polypropylene and ethylene/propylene triple synthetic rubber polymer blends [16], and ethylene–vinyl acetate copolymers (EVACs) with different contents of vinyl acetate (VA) [17]. This made it possible to significantly improve the thermal and mechanical properties of the polymer blends produced by the SEDS process, compared to melt blending. In the present work, in contrast to the mentioned above publications, we, for the first time, used the same SEDS technique for mixing a PVC/PE blend, which are not mixing in the melt, in the whole concentration range from 0 to 100 wt% without the use of compatibilizing additives.

## 2. Materials and Methods

The following polymers were used for the present study: polyvinyl chloride grade ПШC-M (PVC), and linear high-pressure polyethylene grade 5118-QM (HPPE) (supplier PAO “SIBUR Holding”). Carbon dioxide with a purity of 0.99 wt fraction was purchased from Techgasservices (Russia). Organic solvents: toluene with a purity of 0.998 wt. fraction, dichloromethane with a purity of 0.998 wt fraction, and chloroform with a purity of 99.85%, were supplied by the company “Base No. 1 Chemical Reactive” (Russia). Ready-to-use commercially available PVC in the form of granules containing technological additives (including stabilizers and plasticizers) were used. Some properties of the polymers and chemicals used for the present study are presented in Table 1. All chemicals were used as received from the supplier without any further purification.

### Experimental Method

#### SEDS Process

A detailed description of the SEDS technique can be found in numerous publications and reviews (see, for example, [18,19,20,21,22,23,24]). It is well-known that use of SCF media in the processing of polymers leads to new materials with enhanced properties [18,19,20,21,22,23,24,25,26,27,28]. Supercritical fluids in various processes of production can be used as an anti-solvent or precipitant, for example, SEDS processes [19]. The SEDS process is based on the unique solubility properties of supercritical fluids, such as solubility in ordinary solvents and lower solvent power of the solvents for compounds in solution. SEDS is a highly sensitive technology for process parameters (temperature, pressure, and concentration of materials) that make it possible to obtain uniform particles with specific physicochemical properties (with special characteristics) and sizes. In the SEDS method, the initial solid material is dissolved in a conventional organic solvent, and then the solution is brought into contact with a SCF that does not dissolve the solid material. By varying the technological conditions (manipulating the process condition) in the supercritical reactor (pressure, temperature, concentration, vibration, etc.), it is possible to achieve rapid deposition of the initial product in the form of fine particles in the volume. There are a number of modifications of the SCF antisolvent method, such as SAS (Supercritical Anti-Solvent), GAS (Gas Anti-Solvent), SEDS (Solution Enhanced Dispersion by Supercritical Fluids), and ASES (Aerosol Solvent Extraction System); see for example, [11,13,19,22,29,30,31,32]. The difference between these methods is the contact between solution and antisolvent. A detailed description of these methods can be found in series review papers [18,19,22,23,24,25,26,27,28,29,30,31,32,33,34].

In the SEDS process, the liquid solution and SCF are sprayed together using a coaxial nozzle. The SCF, in this process, serves as an antisolvent as a dispersion media. Technologically SEDS process is implemented as follows: the SCF and the liquid solution of the dispersible material are spontaneously contacting by passing through coaxial nozzles and delivering to the particle formation vessel (precipitator), which generates the finely dispersed mixture and promptly forms a microparticle. When solvent and polymer solution came into contact with SCF, rapid recrystallization occurred due to high supersaturation of the mixed solute-solvent and polymer. The processing media (SCF), organic solvent, and solvent and polymer (solute) create operation parameters to control the SEDS process of supercritical particle formation phenomena. Further details can be found in our previous series of publications [15,16,17,28,32].

**Figure 1:**
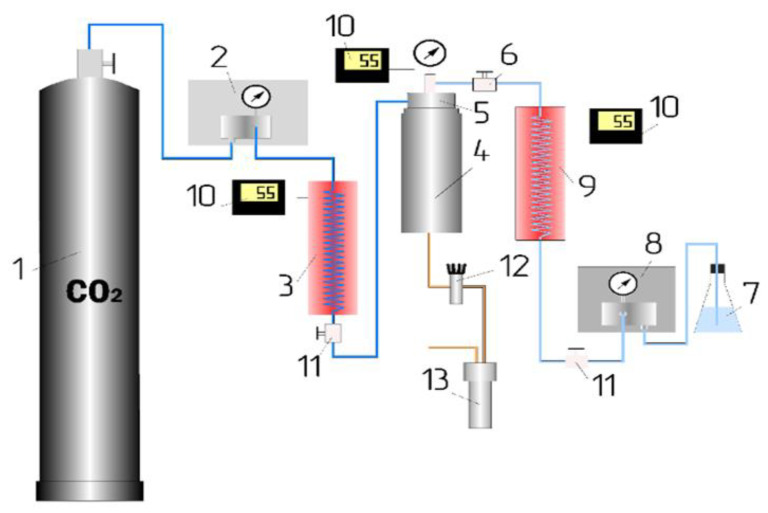
Schematic diagram of the experimental apparatus for supercritical SEDS dispersion of polymer blends: 1—cylinder with CO_2_; 2—CO_2_ supply pump; 3—CO_2_ heater; 4—reactor (precipitation vessel); 5—coaxial nozzle; 6—valve on the solution supply line to the nozzle; 7—container for a solution (test sample + organic solvent); 8—solution supply pump (THAR, USA); 9—solution heater; 10—temperature control; 11—valve; 12—back pressure regulator; and 13—separator.

A schematic diagram of an experimental apparatus designed for SEDS dispersion of polymer blends is shown in Figure 1.

The experimental SEDS dispersion apparatus consists of a system for creating, regulating, and measuring pressure-12 and temperature-10; systems for supplying a solution-8,9 of a mixture of polymers in an organic solvent and an antisolvent; a deposition cell-4; and a microparticle collection system-13.

The experimental details and procedure of measurements were previously described in our several publications [15,16,17]. To supply a solution of a mixture of polymers in an organic solvent-7 and CO_2_-1, plunger pumps from THAR (USA) are used-2,8. The concentration of polymers in an organic solvent is 4 wt%. A one-liter cylindrical stainless-steel container is used as the precipitation cell-4. The pressure in the cell is measured with a pressure gauge and adjusted by the back pressure regulator-12. The injection of liquid solution and the supply of supercritical carbon dioxide occur simultaneously through a coaxial nozzle-5. The process of injection of the solution into the SC CO_2_ occurs within two hours. In this case, a solution of polymers in an organic solvent is fed through the inner hole, and CO_2_ through the outer annular gap. To collect dispersed microparticles, a metal substrate is installed at the bottom of the reactor. The organic solvent remaining after the experiment is collected in the separator-13. The product yield is 98.4% of the mass of the initial polymers.

The polymer blends obtained by the supercritical SEDS dispersion process are analyzed by scanning electron microscopy (SEM), using an AURIGA Cross Beam instrument (Germany) with an INCA X-MAX energy dispersive spectrometer.

The polymers compositions obtained by blending in the melt were prepared in a mixing chamber “Measuring Mixer 350E” of Brabender mixing equipment “Plasti-Corder^®^ Lab-Station” (Germany). The HPPE/PC compositions were mixed for 3 min at a temperature of 155 °C. In all cases, the rotation speed of the rotors during mixing was 60 rpm. After being removed from the mixing chamber, the samples were passed through cold rollers and kept at room temperature for a day to relieve internal stresses.

A differential scanning calorimeter (DSC) DSC-200 TA (USA) with Pyris software has been used to study the kinetics of crystallization and phase transformation in blends of copolymers. The heating and cooling rates were 10 °C/min. The studies were carried out according to the method described in [35]. The solubility of polymers in organic solvents was performed using the well-known technique described in the works [32,36].

The samples for mechanical property test were obtained by pressing on a YT-30RS hydraulic press. The following pressing procedure is implemented: The material is placed in a 100 × 100 mm box on a lavsan fiber support between two compression molds, which are steel sheets. The form assembled in this way with the composite is installed on the lower plate of the press, after which the upper movable plate is lowered to create pressure. Then, preheating is carried out for 5 min. The sample is molded under a pressure of 100 kgf/cm^2^ at a temperature of 175 °C for 5 min, after which cooling is carried out for 30 s without removing the load. The cooling is then turned off, the top plate is raised, and the finished plate is removed from the box.

Tensile strength is determined in accordance with the requirements of ASTM D 882. The test sample is punched out in the form of a blade using a special knife. The resulting blade should have a flat surface without defects. Before testing, the thickness and width of the working part of the samples were measured in 3 places, after which the average value and standard deviations of the thickness and width of the working part of the sample were calculated. The rupture stress at brake is defined as the ratio of the force at which the sample is mechanical breakdown to the cross-sectional area of the working part of the sample before tensile rupture. The determination of the deformation-strength properties of the sample is carried out on a tensile tester TeST GmbH model 112.5 kN (Germany) at a test speed of 50 mm/min. The estimated length of the sample for determining the relative elongation is 20 mm. The test is carried out at a temperature of 20 ± 2 °C and a relative humidity of 50 ± 5%.

## 3. Results and Discussion

The detailed description of the SEDS dispersion procedure is available elsewhere [15,16,17,28,32]. The following SEDS dispersion procedure has been implemented. The SEDS dispersion process has been performed as follows: (1) the initial solid polymers are dissolved in an organic solvent; and (2) then the solution is brought into contact with a supercritical fluid that does not dissolve the solid polymers. By varying the conditions in the reactor (pressure, temperature, vibration, etc.), it is possible to achieve more or less rapid deposition of the initial product in the form of fine particles in the volume or by spraying.

Thus, the implementation of the supercritical SEDS method provides that: (1) no solubility of polymers in supercritical carbon dioxide; (2) polymers should be well soluble in an organic solvent; and (3) the carbon dioxide–organic solvent binary system should be in a single-phase supercritical fluid region. It is well-known [28] that polymers HPPE and PVC do not dissolve in SC carbon dioxide, which justifies the use of the SEDS method.

**Figure 2:**
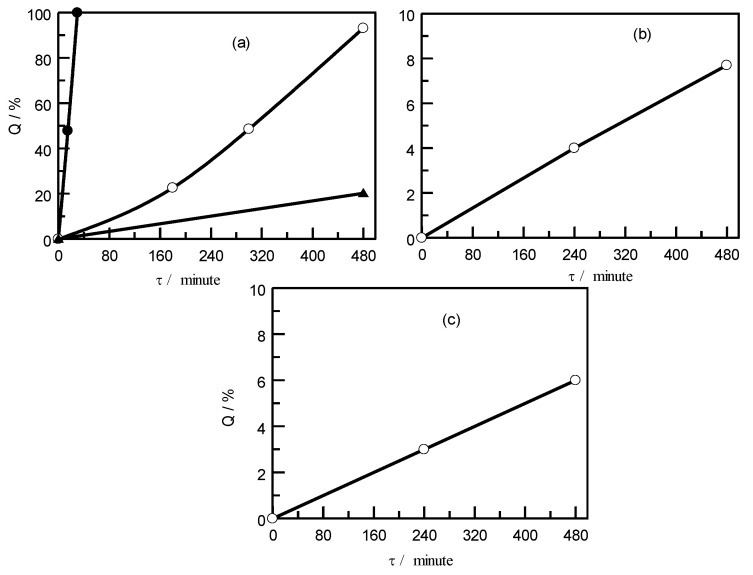
Solubility (Q) of PE in various organic solvents as a function of time at selected temperatures: (**a**) in toluene at ●-*T* = 373 K; ο-*T* = 353 K; and ▲-*T* = 323 K; (**b**) in dichloromethane at *T* = 308.15 K; and (**c**) in chloroform at *T* = 323 K.

Toluene, chloroform, and dichloromethane were used as solvents for measuring the solubility of PE and PVC. When selecting the experimental temperature range for each solvent, their boiling points were taken into account. The results of the solubility study of polyethylene in toluene, chloroform, and dichloromethane are depicted in Figure 2.

The dissolution time of PE in toluene decreases with increasing temperature. For example, the complete dissolution of PE in toluene at a temperature of 353 K occurs within 480 min, while at a temperature of 373 K, complete dissolution is achieved within 30 min.

The polymer mass loss (Q) as a function of time is calculated as

Q = (w_0_ − w_τ_)/w_0_ × 100

where w_0_ is the initial mass of the polymer sample, and w_τ_ is its mass at time τ.

**Figure 3:**
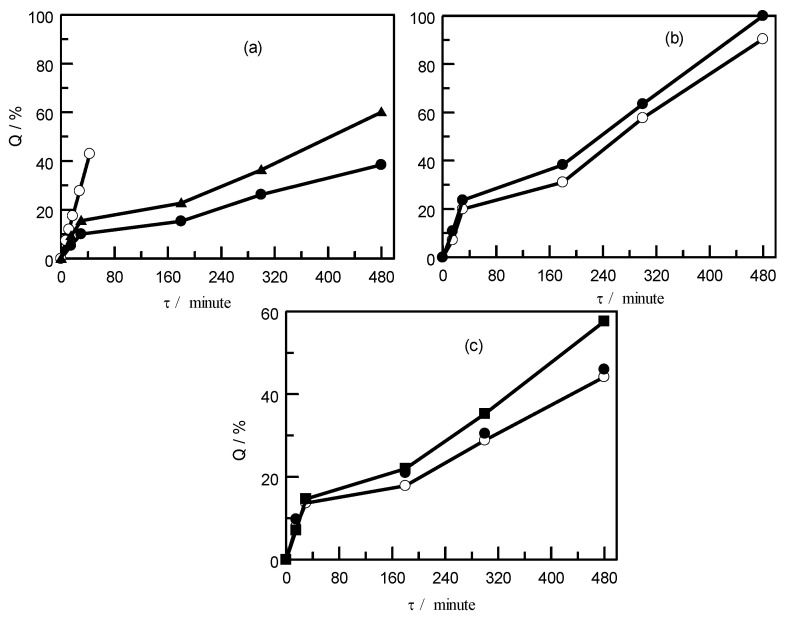
Solubility of PVC in various organic solvents as a function of time at selected temperatures: (**a**) in toluene at ο-*T* = 373 K, ▲-*T* = 353 K, ●-*T* = 323 K; (**b**) in dichloromethane at ●-*T* = 308 K; ο-*T* = 298.15 K; and (**c**) in chloroform at *T* = 298 K; 6-chloroform, ■-*T* = 323 K; ο-*T* = 298 K; ●-*T* = 308 K; 8-chloroform.

For the present study, the use of chloroform and dichloromethane as a solvent is suitable due to long time (480 min) and low solubility of PE (see Figure 2). A study of PVC solubility in the above-mentioned organic solvents showed the following results (see Figure 3). The use of toluene and chloroform as a solvent at the maximum possible temperatures for each of them shows not the best dissolution in comparison with dichloromethane. For example, toluene and chloroform within 480 min dissolve 60.1% at a temperature of 373 K, and 57.6% at a temperature of 323 K, respectively. Almost complete dissolution with dichloromethane at a temperature of 308 K is achieved within 480 min. For a similar time, at a temperature of 298 K, 90% PVC is dissolved.

The present results are consistent with the theory of polymer solutions [37], and are determined by the experimentally obtained solubility parameters of polymers and solvents shown in Table 2. As one can see from Table 2, the closer the solubility parameters of the solvents and polymers, the higher the mutual solubility.

The relatively high solubility of HPPE and PVC in organic solvents confirms the high productivity and energy efficiency of the SEDS dispersion process.

In order to successfully perform the supercritical fluid antisolvent (SEDS) deposition process, the characteristics of the phase equilibrium (VLE) of the system of organic solvent and antisolvent system are required to identify the supercritical region (parameters) for a given mixture (CO_2_—toluene/dichloromethane).

Based on the solubility of polymers in organic solvents data, toluene and dichloromethane were selected for the present study. Since polymers blend according to the SEDS method, it is necessary to prepare a mixture of polymers in an organic solvent, and we are interested in a mixture of solvents. The results of an experimental study of the phase equilibrium of the CO_2_—toluene/dichloromethane system are presented in our previous work [15]. According to the VLE data reported in our previous publication [15] for CO_2_—toluene/dichloromethane, the thermodynamic system CO_2_—toluene/dichloromethane demonstrates type I and II phase behavior. A single-phase supercritical fluid state corresponding to the method of SEDS dispersion, which is supposed to be implemented at *T* = 313 K, takes place at pressures below 8.0 MPa.

Thus, the preferred operating parameters for the implementation of the SEDS process for PVC/PE 5118 polymer blends were defined (Table 3).

**Figure 4:**
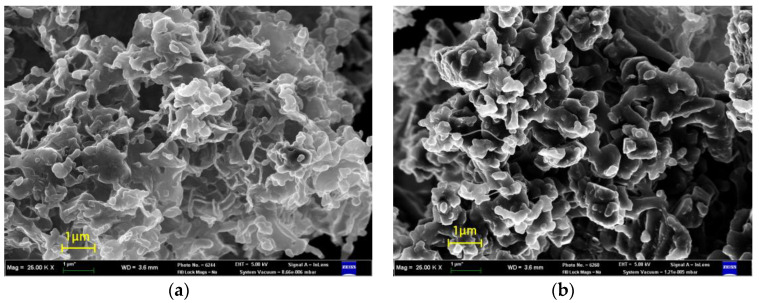

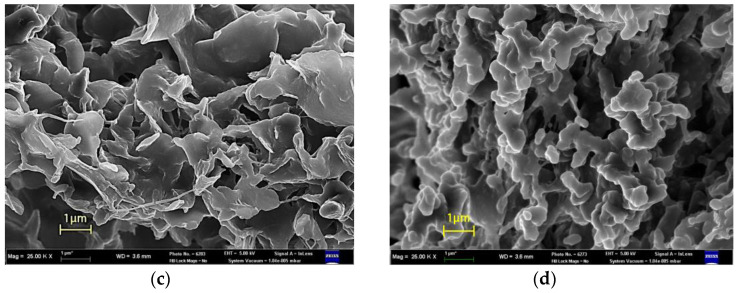
SEM image (morphology) of PVC (50%)/HPPE(50%) blend, produced by SEDS dispersion at various temperatures and pressures: (**a**)—*T* = 313 K, *P* = 8 MPa; (**b**)—*T* = 313 K, *P* = 15 MPa; (**c**)—*T* = 313 K, *P* = 25 MPa; and (**d**)—*T* = 333 K, *P* = 15 MPa.

Figure 4 shows SEM images of a PVC (50%)/HPPE (50%) polymer blend produced at various pressures and temperatures. As Figure 4 shows, the obtained particles have an irregular porous shape and coalescence has occurred. Because the injected polymer solution was in contact with supercritical CO_2_ immediately after exiting the nozzle, the formation of fine droplets of the polymer solution was difficult. In this situation, nucleation and growth occurred simultaneously in solution, thus forming an interconnected structure of particles. It can be seen that the particle sizes of the mixture are larger in samples obtained at high pressures, which occurs due to an increase in the degree of coalescence. The difference in the degree of coalescence is probably associated with an increase in the ability of supercritical CO_2_ to extract an organic solvent due to an increase in CO_2_ density.

**Figure 5:**
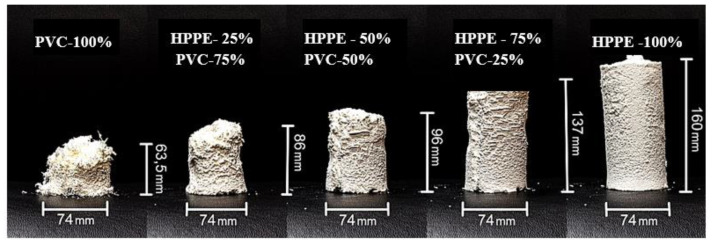
Composite materials with different content of HPPE and PVC produced using the SEDS method.

Figure 5 shows the polymer composite materials obtained with different contents of HPPE and PVC.

**Figure 6:**
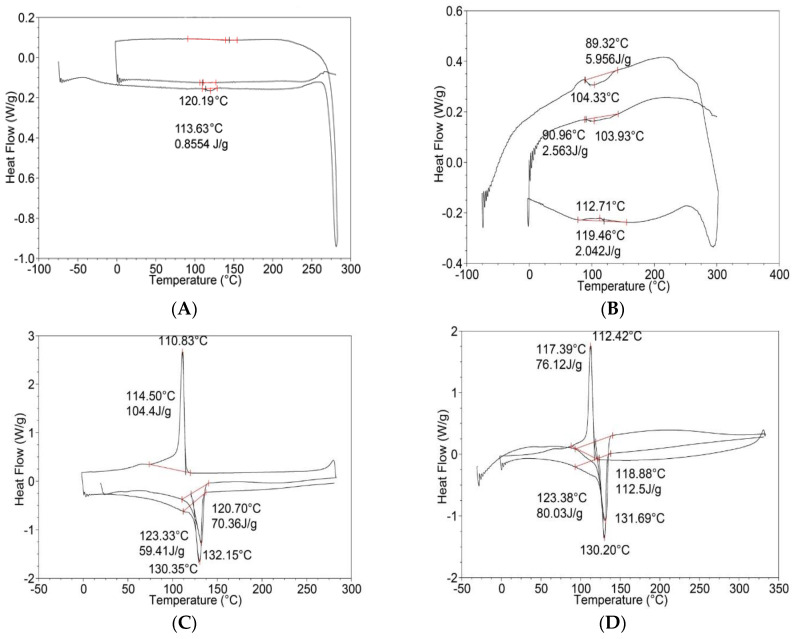

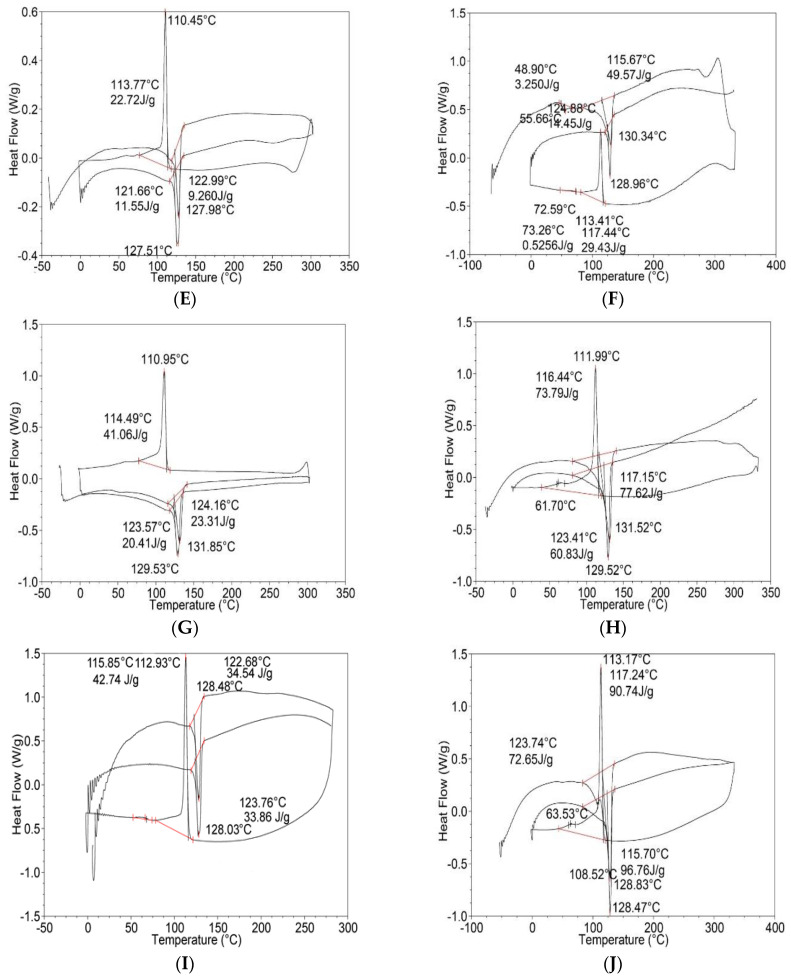
(**A**–**J**). DSC scan diagram of melting-crystallization-melting process of the pure PVC polymers: (**A**) original commercial sample; (**B**) obtained by SEDS method); pure HPPE polymers; (**C**) original sample; (**D**) obtained by SEDS method); and polymer blends; (**E**) 25% HPPE/75% PVC (blending in melt); (**F**) 25%HPPE/75% PVC obtained by the SEDS method; (**G**)-50% HPPE/50% PVC obtained by blending in melt; (**H**) 50% HPPE/50% PVC obtained by the SEDS method; (**I**) 75% HPPE/25% PVC obtained by blending in melt; and (**J**) 75% HPPE/25% PVC obtained by the SEDS method. The effect of HPPE/PVC ratio on the thermal characteristics of the polymer blends.

Polymer samples obtained by SEDS and blending in the melt were analyzed using a differential scanning calorimeter (DSC). Before studying the melting and crystallization processes of HPPE/PVC polymer blends obtained both by blending in the melt and in supercritical carbon dioxide (SEDS process), the DSC diagrams of the original pure components HPPE and PVC were analyzed. The results of the DSC of HPPE/PVC polymer blends and its pure components produced by SEDS and blending in the melt are depicted in Figure 6A–J. As the DSC experiment shows (Figure 6A), the melting-crystallization-melting diagram of the original pure PVC exhibits a very small peak at a temperature of 120.19 °C, with a heat of fusion of 0.85 kJ/kg, which indicates an absence of crystalline phase, since commercial PVC is an amorphous polymer. During the cooling run, no significant peaks and inflections are observed, which indicates the absence of the PVC crystallization process. Reheating also shows no change in the DSC curve behavior, which confirms the absence of a crystalline phase.

In the melting diagram of PVC obtained by the SEDS method (see Figure 6B, at an experimental condition of 313.15 K and 8 MPa, Table 3), we found one melting peak at a temperature of 104.33 °C with a heat of fusion of 5.95 kJ/kg, which confirmed the presence of a crystalline phase. Upon cooling, a small crystallization peak is observed with a heat of 2.04 kJ/kg at a temperature of 112.71 °C. The repeated heating, one melting peak, is also observed at a temperature of 103.93 °C, but the heat of fusion decreased to 2.56 kJ/kg, which indicates a decrease in the degree of PVC crystallinity. However, compared to the original commercial PVC, there is a crystalline phase. In the melting-crystallization-melting diagram of the initial HPPE (Figure 6C), we observed one melting peak at a temperature of 132.15 °C with a heat of fusion of 70.36 kJ/kg. During crystallization, one peak is observed at a temperature of 110.83 °C. When reheating, one melting peak is also observed at a temperature of 130.35 °C, while the heat of fusion is 59.41 kJ/kg, which is lower than the value of the initial HPPE polymer. This can be explained by less equilibrium cooling conditions. The melting-crystallization-melting DSC diagram of the initial HPPE polymer obtained by the SEDS method (see Figure 6D, at 313.15 K and 8 MPa), found one melting peak at a temperature of 131.69 °C with a heat of fusion of 112.5 kJ/kg, which exceeds 1.6 times the heat of fusion of the original polymer. During crystallization, one peak is observed at a temperature of 117.39 °C with the heat of crystallization of 76.39 kJ/kg. Upon reheating, single melting peak is also observed at a temperature of 130.2 °C, while the heat of fusion is 80.03 kJ/kg, which is close to the value of the original polymer. It should be noted that the SEDS process increases the heat of fusion of HPPE in comparison with the original commercial polymer sample. This indicates an increase in the degree of crystallinity. For PVC, it can be noted that during the SEDS process, even the appearance of a crystalline phase is observed, which improves the polymer characteristics.

The DSC melting-crystallization-melting curves of HPPE/PVC polymer blends with compositions of 25, 50 and 75 mass % obtained by blending in the melt and SEDS processes have been studied (see Figure 6C–J). Figure 6E shows the DSC diagram of a 25% HPPE/75% PVC polymer blend obtained by blending in the melt. As can be seen, only a single melting peak at a temperature of 127.98 °C with a heat of fusion of 9.26 kJ/kg, corresponding to the HPPE phase, is observed. Moreover, the heat of fusion is lower than the additive value 17.57 kJ/kg, which is due to the influence of the PVC phase on the crystallization of polyethylene. During the crystallization of the same HPPE/PVC blend, only one peak is observed at a temperature of 110.45 °C, with a heat of fusion of 22.72 kJ/kg, which also corresponds to the HPPE phase. Upon reheating, one peak is also observed at a temperature of 127.51 °C with a heat of fusion of 11.55 kJ/kg, which is also below the additive value and belongs to the HPPE phase. The melting DSC diagram of the same polymer blend obtained by the SEDS method (Table 3, at 313.15 and 8 MPa) is shown in Figure 6F. In this case, two melting peaks at a temperature of 48.9 °C with a heat of fusion of 3.25 kJ/kg, corresponding to the PVC phase, and the crystalline phase at a temperature of 130.34 °C with a heat fusion of 49.57 kJ/kg, corresponding to the HPPE phase have been found. The heat of fusion 17.57 kJ/kg is much higher than the additive value. During the crystallization process of the polymer blends, a single peak is observed at a temperature of 117.44 °C, with a released heat of 29.43 kJ/kg, which also corresponds to the HPPE phase. Upon reheating, only one peak is observed at a temperature of 128.39 °C with a heat of fusion of 14.45 kJ/kg, which is approximately equal to the additive value and also belongs to the HPPE phase. It can be concluded that the polymer blend obtained by the SEDS method is more completely crystallizing. This can be explained by the fact that the heat of fusion exceeds the additive values, and when reheated, the blend behaves as usual, like blending in the melt.

The melting-crystallization-melting curve (Figure 6G) of a polymer blend of 50% HPPE-50% PVC, obtained by blending in the melt, illustrates the presence of a single melting peak at a temperature of 131.23 °C with a heat of fusion of 23.31 kJ/kg, which is related to the HPPE phase; moreover, is lower than the additive value of 35.15 kJ/kg. During the crystallization of the polymer mixture, a single peak is observed at a temperature of 110.95 °C, with a released heat of fusion of 41.06 kJ/kg, which also corresponds to the HPPE phase. Upon reheating, one peak is also observed at a temperature of 129.53 °C with a heat of fusion of 20.41 kJ/kg, which is also lower than the additive values and belongs to the HPPE phase. As can be noted, the presence of the PVC phase prevents the HPPE crystallization process. The melting-crystallization-melting curve of a polymer mixture of 50% HPPE-50% PVC obtained by the SEDS method (Table 3, at 313 K and 8 MPa) is depicted in Figure 6H. As can be seen, only one melting peak is observed at a temperature of 131.32 °C with a heat of fusion of 77.62 kJ/kg, corresponding to the HPPE phase. The heat of fusion is more than two times higher than the additive value of 35.15 kJ/kg. During the crystallization process of the polymer blend, one peak is observed at a temperature of 111.99 °C, with a released heat of 73.79 kJ/kg, which also corresponds to the HPPE phase. Upon repeated heating, one peak is also observed at a temperature of 129.52 °C with a heat of fusion of 60.83 kJ/kg, which is also higher than the additive value and belongs to the HPPE phase. This means that a structure with a high degree of order is retained even after thermal treatment.

The study of the DSC curve (Figure 6I) of the third polymer blend of 75% HPPE/25% PVC showed that when blending in the melt, one peak is observed at a temperature of 128.48 °C with a heat of fusion of 34.54 kJ/kg. The peak corresponds to the HPPE phase, which is lower than the additive value of 52.72 kJ/kg. During the crystallization of the polymer blend, one peak is observed at a temperature of 112.93 °C, with the heat of crystallization at 42.74 kJ/kg, which also corresponds to the HPPE phase. Upon reheating, one peak is also observed at a temperature of 128.03 °C with a heat of fusion of 33.86 kJ/kg, which is also lower than the additive value and associated with the HPPE phase. Thus, the presence of the PVC phase prevents the HPPE crystallization process. The opposite results were observed for the DSC curve of the same polymer blend (75% HPPE/25% PVC) produced by the SEDS method (see Figure 6J at 313.15 K and 8 MPa). In this case single peak at a temperature of 128.84 °C with a heat of fusion of 96.76 kJ/kg, which is attributed to the HPPE phase. The heat of fusion is two times higher than the additive value of 52.72 kJ/kg. During the crystallization of the mixture, one peak is observed at a temperature of 113.17 °C, with a released crystallization heat of 90.74 kJ/kg, which also corresponds to the HPPE phase. Single peak has also been observed at a temperature of 128.47 °C with a heat of fusion of 72.65 kJ/kg upon repeated heating. The value of the heat of fusion is also higher than the additive one and is related to the HPPE phase. This means that a structure with an increased degree of order is retained even after heat treatment. The derived results on the DSC melting curves of polymer blends HPPE/PVC are summarized in Table 4.

As can be seen, for all mixtures and pure polymers obtained by the SEDS method, the specific heat of fusion exceeds the values obtained by blending in the melt by 1.5–5 times. Therefore, the best Δ_fus_*H* thermodynamic conditions for the crystallization process are created in the case of blending by the SEDS method. This means that the specific heat of fusion is higher than the additive value and, accordingly, the degree of crystallinity is higher, and this effect persists after heat treatment. An increase in the degree of crystallinity should lead to an improvement in the physical and mechanical properties.

In the present work, we studied the physical and mechanical characteristics of the polymer blends produced by SEDS and blending in the melt techniques. The study of the physical and mechanical characteristics was carried out on pressed samples according to the methods presented in the experimental part (see above). The measured property data are summarized in Table 5.

As can be seen from Table 5, for most of the studied blends, the physical and mechanical characteristics of the compositions obtained by blending using the SEDS method exceed those of the blends produced by blending in the melt, especially with regard to tensile strength. With regard to relative elongation at break, lower values for blends obtained by the SEDS method can be explained by the fact that industrial PVC contains plasticizers, which are washed out during their production. As a result, the relative elongation drops for the entire mixture, but the tensile strength increases significantly.

## 4. Conclusions

New experimental data related to the solubility of PVC and HPPE in organic solvents (toluene, dichloromethane, and chloroform) over the temperature range from 298.15 to 373.15 K are reported. The supercritical SEDS dispersion of PVC and HPPE polymer blends at pressures between 8.0 and 25 MPa and at temperatures from 313 to 333 K are reported in the present work. The kinetics of crystallization and phase transformation in polymer blends obtained by blending in a melt and using the supercritical SEDS method have been studied by the DSC technique.

The thermal (fusion temperature, *T*_fus_, and heat of fusion,Δ_fus_*H*) and mechanical characteristics (strength and relative elongation) of the polymer blends produced by SEDS and blending in the melt techniques have been studied. For all polymer blends and pure polymers obtained by the SEDS method, the heat of fusion, Δ_fus_*H* exceeds the values obtained by blending in the melt by 1.5 to 5 times. Therefore, the best thermodynamic conditions for the polymer blended crystallization process are created in the case of blending by the SEDS method. This means that the heat of fusion of the obtained polymer blends is higher than the additive value and, accordingly, the degree of crystallinity is higher, and this effect persists after heat treatment. An increase in the degree of crystallinity should lead to an improvement in the physical and mechanical properties of the produced polymer blends. The thermal and mechanical characteristics of the polymer compositions obtained by blending using the SEDS method exceed those of the blends produced by blending in the melt, especially with regard to tensile strength. The relative elongation decreases for the all-polymer blends, but their tensile strength increases significantly.

## Figures and Tables

**Table 1:** Physical properties of polymers and samples description studied in this work.

Polymers	Melting Temperature (*T*_m_)/K		Heat of Fusion (Δ_fus_*H*)/kJ∙kg^−1^		
PVC	*T*_m_ = 387.04 K		0.650		
HPPE	*T*_m_ = 405.30 K		70.36		
Samples	*M* (kg·kmol^−1^)	CAS	Source	Purity (m.f.)	H_2_O Content ^a^
Carbon dioxide	44.010	124-38-9	TechGasServ	>0.990	65 ppm
Toluene	92.138	108-88-3	Chem Reactive-1	>0.998	<1000 ppm
Dichloromethane	84.933	75-09-2	Chem Reactive-1	>0.998	<1000 ppm
Chloroform	119.38	67-66-3	Chem Reactive-1	>0.9985	<1000 ppm

^a^ Karl Fischer method. The suppliers furnished purity of the samples.

**Table 2:** Solubility parameter of polymers and solvents [38].

Solvents and Polymers	Solubility Parameter, (cal/cm^3^)^1/2^
Toluene	8.97
Chloroform	9.30
Dichloromethane	9.95
HPPE	7.94
PVC	9.57

**Table 3:** Operation parameters of the process of dispersing polymer blends by the SEDS method (concentration of polymers blend in a solvent of 4 wt%; nozzle diameter 200 μm).

Polymers	*T*, K	*P*, MPa
PVC (100%)	313	8
HPPE (100%)	313	8
PVC (75%)/HPPE (25%)	313	8
PVC (50%)/HPPE (50%)	313	8
PVC (25%)/HPPE (75%)	313	8
PVC (50%)/HPPE (50%)	313	15
PVC (50%)/HPPE (50%)	313	25
PVC (50%)/HPPE (50%)	333	15

**Table 4:** Summary of the DSC melting curves of polymer blends HPPE/PVC at (*T* = 313 K and *P* = 8 MPa) ^a^.

Polymer Blends	Blending in the Melt		Blending by SEDS Method	
	*T*_fus_, °C	Total Δ_fus_*H*, kJ/kg	*T*_fus_, °C	Total Δ_fus_*H*, kJ/kg
HPPE (100%)	132.15 ± 0.02	70.36 ± 0.02	131.69 ± 0.02	112.5 ± 0.02
PVC (100%)	120.19 ± 0.02	0.85 ± 0.02	104.33 ± 0.02	5.95 ± 0.02
HPPE (25%) PVC (75%)	127.98 ± 0.02	9.26 ± 0.02	48.90 ± 0.02 130.34 ± 0.02	3.25 ± 0.02 49.57 ± 0.02
HPPE (50%) PVC (50%)	131.23 ± 0.02	23.31 ± 0.02	131.32 ± 0.02	77.62 ± 0.02
HPPE (75%) PVC (25%)	128.48 ± 0.02	33.49 ± 0.02	128.47 ± 0.02	108.52 ± 0.02

^a^ Standard absolute *u* uncertainties (0.95 level of confidence) are: *u*(*T*_fus_) = 0.01 °C; *u*(Δ_fus_*H*) = 0.01 kJ·kg^−1^.

**Table 5:** Mechanical properties (tensile strength and relative elongation) of produced polymer blends using SEDS method and blending in the melt ^a^.

Polymer Blends	Polymer Blend Obtained by SEDS Method at (*T* = 313 K и *P* = 8 MPa)		Polymer Blend Obtained by Blending in the Melt	
	Tensile Strength, (σ_p_), MPa	Relative Elongation (ε), %	Tensile Strength, (σ_p_), MPa	Relative Elongation (ε), %
PVC-75% HPPE-25%	10.66 ± 0.02	2.40 ± 0.03	2.32 ± 0.02	155.0 ± 2
PVC-50% HPPE-50%	9.22 ± 0.02	12.80 ± 0.18	5.08 ± 0.02	370.0 ± 5
PVC-25% HPPE-75%	10.35 ± 0.02	486.7 ± 7	12.91 ± 0.02	820.0 ± 11
HPPE-100%	17.39 ± 0.02	790.0 ± 11	15.34 ± 0.02	616.7 ± 8
PVC-100%	28.71 ± 0.02	3.90 ± 0.05	4.27 ± 0.02	296.7 ± 4

^a^ Standard absolute *u* and relative $u_{r}$ uncertainties (0.95 level of confidence) are: *u* (σ_p_) = 0.01 MPa; $u_{r}$ (ε) = 1.4%.

## Data Availability

Not applicable.
